# Multi-year molecular quantification and ‘omics analysis of *Planktothrix*-specific cyanophage sequences from Sandusky Bay, Lake Erie

**DOI:** 10.3389/fmicb.2023.1199641

**Published:** 2023-06-29

**Authors:** Katelyn M. McKindles, Makayla Manes, Michelle Neudeck, Robert Michael McKay, George S. Bullerjahn

**Affiliations:** ^1^Ecology and Evolutionary Biology, University of Michigan, Ann Arbor, MI, United States; ^2^Great Lakes Institute for Environmental Research, University of Windsor, Windsor, ON, Canada; ^3^Great Lakes Center for Fresh Waters and Human Health, Bowling Green State University, Bowling Green, OH, United States; ^4^Department of Microbiology, The Ohio State University, Columbus, OH, United States

**Keywords:** cyanophage, *Planktothrix agardhii*, PaV-LD, metagenome, metatranscriptome, qPCR

## Abstract

**Introduction:**

*Planktothrix agardhii* is a microcystin-producing cyanobacterium found in Sandusky Bay, a shallow and turbid embayment of Lake Erie. Previous work in other systems has indicated that cyanophages are an important natural control factor of harmful algal blooms. Currently, there are few cyanophages that are known to infect *P. agardhii*, with the best-known being PaV-LD, a tail-less cyanophage isolated from Lake Donghu, China. Presented here is a molecular characterization of Planktothrix specific cyanophages in Sandusky Bay.

**Methods and Results:**

Putative *Planktothrix*-specific viral sequences from metagenomic data from the bay in 2013, 2018, and 2019 were identified by two approaches: homology to known phage PaV-LD, or through matching CRISPR spacer sequences with *Planktothrix* host genomes. Several contigs were identified as having viral signatures, either related to PaV-LD or potentially novel sequences. Transcriptomic data from 2015, 2018, and 2019 were also employed for the further identification of cyanophages, as well as gene expression of select viral sequences. Finally, viral quantification was tested using qPCR in 2015–2019 for PaV-LD like cyanophages to identify the relationship between presence and gene expression of these cyanophages. Notably, while PaV-LD like cyanophages were in high abundance over the course of multiple years (qPCR), transcriptomic analysis revealed only low levels of viral gene expression.

**Discussion:**

This work aims to provide a broader understanding of Planktothrix cyanophage diversity with the goals of teasing apart the role of cyanophages in the control and regulation of harmful algal blooms and designing monitoring methodology for potential toxin-releasing lysis events.

## 1. Introduction

Cyanobacterial harmful algal bloom (cHAB) prevalence is increasing worldwide due to a combination of nutrient pollution and climate change (O’Neil et al., 2012; Wurtsbaugh et al., 2019; Gobler 2020). These blooms are of concern due to their ability to impact human health, ecosystem processes, and regional economies. cHABs can produce multiple toxins and secondary metabolites which have been linked to neurotoxicity, respiratory distress, cancers with prolonged exposure, and death of livestock and pets (Codd et al., 2020; Metcalf et al., 2021; Wang et al., 2021; Mchau et al., 2022). They can grow so dense that they block light penetration in aquatic ecosystems, leading to a reduction in the diversity and abundance of other species (Sunda et al., 2006). Further, cHABs can cause significant economic losses in affected regions by reducing the value of aquatic products, reducing tourism, and reducing home values (Hoagland et al., 2002; Sanseverino et al., 2016; Guillotreau et al., 2021; Heil and Muni-Morgan, 2021; Wolf et al., 2022).

*Planktothrix agardhii* can form blooms inhabiting eutrophic freshwaters worldwide (Kurmayer et al., 2015). *P. agardhii* is of particular concern in the Laurentian Great Lakes region due to annual recurrence in Sandusky Bay, a drowned river mouth draining into the open waters of Lake Erie (Davis et al., 2015; Hampel et al., 2019). Unlike *Microcystis* which dominates in the western basin of Lake Erie, *Planktothrix* grows at a broader temperature range (Foy et al., 1976; Post et al., 1985), is adapted to lower light levels (Oberhaus et al., 2007) and can scavenge ammonium more effectively (Hampel et al., 2019), all of which allow it to thrive in turbid Sandusky Bay, in which nitrogen can become limiting due to high rates of denitrification (Salk et al., 2018).

Previous work has sought to better understand the dominance of *Planktothrix agardhii* in Sandusky Bay through metagenome and metatranscriptome analysis (Hampel et al., 2019; McKindles et al., 2022). Part of this characterization included an analysis of the host foreign DNA defense response, the CRISPR-cas system, which was used to aid in the identification of viral signatures (McKindles et al., 2022). This study noted that only a small percentage of spacer sequences could be identified as previously isolated cyanophage sequences, indicating the presence of heretofore unexplored viral diversity.

Part of the issue of identification of cyanophages is the lack of available reference sequences due to a low success rate in cyanophage isolation. Currently, there is only one isolated cyanophage that is known to target *P. agardhii*: PaV-LD (Gao et al., 2009). This virus belongs to the Podoviridae and was isolated from Lake Donghu (East Lake), China, with a burst size of 340 viral particles per cell and an incubation period of 6–8 days (Gao et al., 2009). PaV-LD contains a double-stranded DNA genome of 95.3 kbp and approximately 142 open reading frames (Gao et al., 2012a). Notably, the phage does not cause complete lysis of the host in the lab, which could be a combination of viral infection strategies and host defense response. Indeed, infections varied depending on stage of host growth, leading to the hypothesis that infections in stable environments were limited due to more stable cell wall structures and more robust host restriction (Gao et al., 2012b).

Despite these studies, there has been little progress to understand the geographical distribution of related *Planktothrix* specific cyanophages in regions where *Planktothrix*-dominated blooms are common, nor is it well known what kind of cyanophage diversity is found in these same regions. To address this, methodology was utilized similar to Morimoto et al. (2020) to identify novel viral signatures in metagenomic data sets from local *Planktothrix agardhii* CRIPSR-cas spacer sequences and then to utilize metatranscriptomics to confirm viral gene expression. Further, PaV-LD major capsid protein (ORF073) primers were utilized to evaluate PaV-LD-like cyanophage presence over the course of 5 bloom seasons in Sandusky Bay, showcasing the high frequency in which viral signatures can be found over the course of a bloom and the consistency in host to virus gene copy ratio across years in the host assocated fraction.

## 2. Materials and methods

### 2.1. Sampling sites and processing

**Figure 1:**
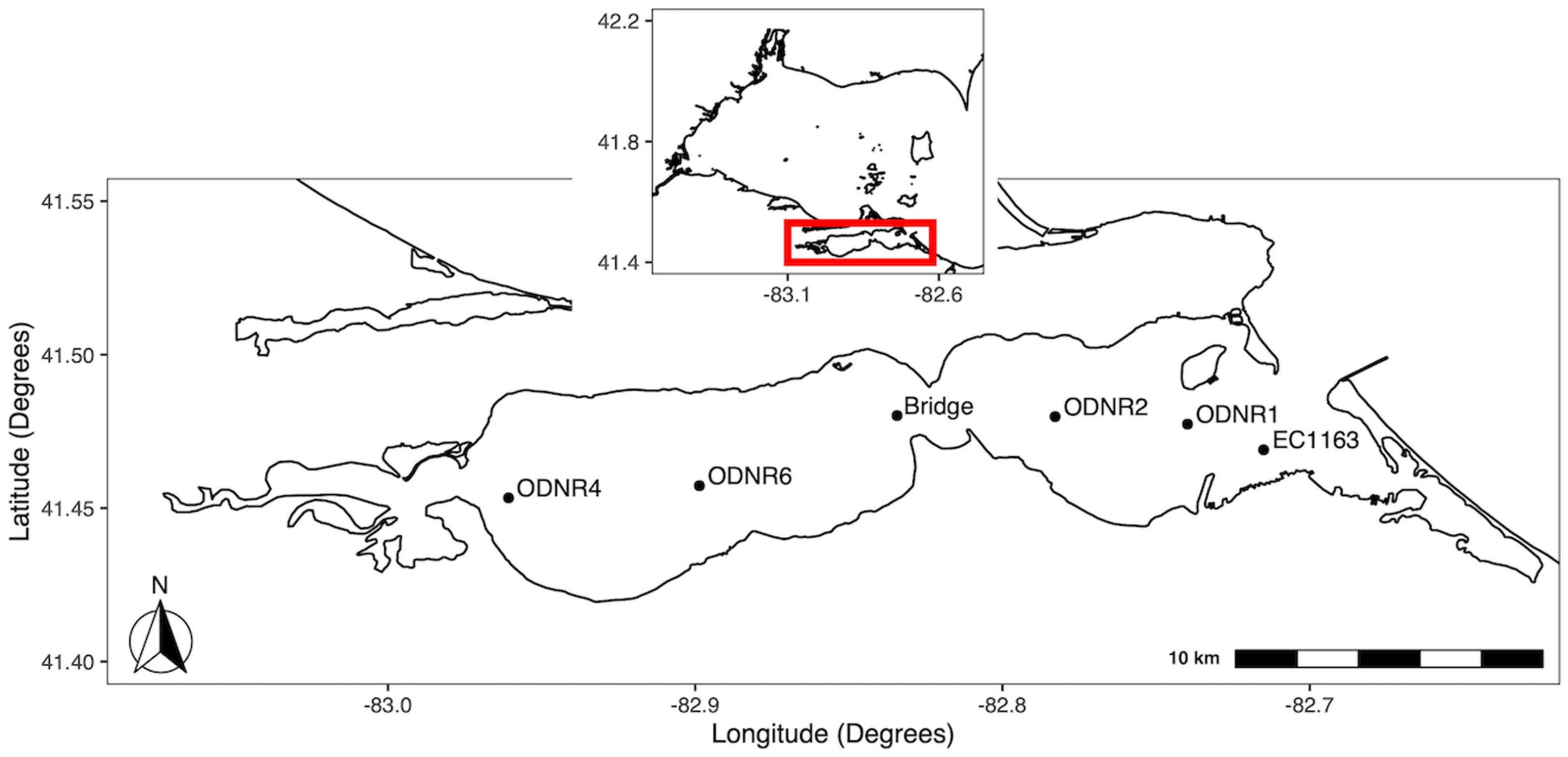
Map of sampling locations in Sandusky Bay. The inset shows the location of Sandusky Bay in relation to the western basin of Lake Erie. Sites were chosen to provide representation of the width and depth of the bay. Sites were ODNR4 (41.453333, −82.960767), Edison bridge (41.480156, −82.834328), ODNR1 (41.477367, −82.739783), and EC1163 (41.469000, −82.715000).

At the southeast region of the western Lake Erie is the shallow (mean depth, 2.6 m), hourglass-shaped Sandusky Bay, which is divided into an outer bay (eastern half) and an inner bay (western half) (Figure 1). In coordination with the Ohio Department of Natural Resources, biweekly water quality surveys of Sandusky Bay were conducted from 2015–2019. In brief, samples were collected during the bloom season (May to October) from three sites between 2015 and 2017 (ODNR4, ODNR1, and EC1163) with the addition of a fourth site, the Edison Bridge, in 2018 and 2019.

Samples were collected in the field from a constant depth of 1 m and biomass was concentrated onto 0.22 μm Sterivex cartridge filters, which were immediately flash-frozen on dry ice. Once in the lab, the filters were stored in a − 80°C freezer until nucleic acids were extracted. In addition to sampling for planktonic biomass, physicochemical data were measured at each site using a model 600 QS water quality probe (YSI Inc., Yellow Springs, OH) capturing surface water temperature, dissolved oxygen concentration, and conductivity (Bullerjahn and McKay, 2020a,b).

During the field season of 2018 and 2019, samples were also collected to measure extracellular PaV-LD presence. One liter of unfiltered water was used in the filtration protocol to collect extracellular phage particles as detailed by Haramoto et al. (2005). Briefly, the whole/unfiltered water was split into two 500-ml duplicate portions for each date and prefiltered through a Whatman 4 filter (GE Healthcare Bio-Sciences, Pittsburgh, PA) to remove large particles. A 0.45-μm pore size S-Pak membrane filter (MilliporeSigma, Burlington, MA) was charged with 250 mM AlCl_3_ and placed on a vacuum filtration apparatus. The filtrate from the first step was passed through the charged S-Pak membrane filter, with the aluminum promoting retention of the cyanophage present in the sample. The filter was washed with 0.5 mM H_2_SO_4_ to remove remaining aluminum ions prior to phage elution with 10 mM NaOH, and neutralization with 100 × TE buffer [1 M Tris–HCl, 100 mM EDTA (pH 8.0)]. Viral concentrates were stored at −80°C until undergoing DNA extraction.

### 2.2. DNA and RNA extraction

DNA was extracted from Sterivex cartridge filters with the DNeasy PowerWater Sterivex DNA isolation kit (Qiagen, Germantown, MD) following the manufacturer’s instructions. DNA quantity was checked using a Quantus Fluorometer (Promega, Madison, WI) and the associated QuantiFluor ONE dsDNA System kit (Promega), per manufacturer’s instructions. RNA was extracted from Sterivex cartridge filters using the Qiagen RNeasy PowerWater Kit by first removing the Sterivex filter from the cartridges under sterile conditions in a laminar flow hood, then following the manufacturer’s instructions.

The xanthogenate-SDS extraction method used for viral filtrate DNA extractions was outlined by Tillett and Neilan (2000) to extract the DNA from environmental samples containing cyanobacteria and was later modified by Yoshida et al. (2006) for use on *Microcystis* cyanophages. Concentrated viral filtrate (200 μL) was added to 750 μL of XS buffer [1% potassium ethyl xanthogenate, 100 mM Tris–HCl (pH 7.4), 20 mM EDTA (pH 8), 1% sodium dodecyl sulfate, 800 mM ammonium acetate] and incubated at 70°C for 30 min, vortexing every 10 min. After incubation for 30 min on ice, isopropanol was added to each tube to 50% (vol/vol). The tubes were incubated at room temperature for a minimum of 10 min followed by centrifugation to pellet the DNA at 12,000 × *g* for 10 min. DNA was washed with 70% ethanol and then air dried for 24 h before resuspension in TE buffer [10 mM Tris–HCl, 1 mM EDTA (pH 8)]. Once the DNA was extracted, it was stored at −80°C for later use.

### 2.3. Monitoring of PaV-LD presence by quantitative PCR

Quantification of *Planktothrix agardhii* occurred as described in McKindles et al. (2021). In brief, *Planktothrix agardhii* species-specific primers rpoC1_Plank_F271 and rpoC1_P_agardhii_R472 were used (Churro et al., 2012; Table 1), which have a primer efficiency of 0.971. The g-block standard was also previously described, generating a standard curve range of 38.66–3.866 × 10^9^ copies μL^−1^.

**Table 1:** Primers sets and g-block standards for *Planktothrix agardhii* and PaV-LD.

Target organism	Target gene name	Forward primer (5′-3′)	Reverse primer (5′-3′)	Product size (bp)	Efficiency	Ref.
All *Planktothrix agardhii*	rpoC1	TGTTAAATCCAGGTAACTATGACGGCCTA	GCGTTTTTGTCCCTTAGCAACGG	224	0.971	McKindles et al. (2021), Churro et al. (2012)
*PaV-LD*	ORF_073RMajor Capsid Protein	GTTAGTCGGATGGGCGAG	CGGGTGGGAGCTAAACCAAT	443	0.9966	This study

To quantify the presence of PaV-LD related cyanophages, primers were generated based on the major capsid protein gene sequence (PaVLD_ORF073R; YP_004957346.1). The capsid gene sequence was uploaded to the primer designer web application (OligoPerfect Primer Designer; Thermo Fisher Scientific, Waltham, MA). Selected primer sets were then analyzed by BLASTn against the nonredundant (nr) database to assess possible spurious hybridization and ensure that the primers were specific to the template, and no other sequences in the nr database could be amplified by the primer sets (Ye et al., 2012). Once tested, the region inclusive of the primer set and extra base pairs in either direction was extracted to create external standards (Supplementary Table S1). External standards were used to determine copy numbers of each qPCR target by creating a 10-fold dilution series of G-block gene fragments (Integrated DNA Technologies, Coralville, IA). G-blocks were diluted to 10 ng μL^−1^ stocks, and the total copy number of G-block fragments was calculated using the formula: number of copies (molecules) = (A ng × 6.0221 × 10^23^ molecules mole^−1^) / ((N × 660 g mole^−1^) × 1 × 10^9^ ng g^−1^), where A is the amount of amplicon in ng, N is the length of the dsDNA amplicon, and 660 g mole^−1^ is the average mass of 1 bp dsDNA (Prediger, 2013). The range for the PaV-LD capsid standard was 20.56–2.056 × 10^9^ copies μL^−1^.

Real-time PCR was performed using 5 μL of each extracted DNA with the PowerUp SYBR Green Master Mix (Thermo Fisher Scientific) and 400 nM of each primer (Table 1). Each sample was amplified under the same conditions multiple times using the different primer sets, as each reaction was a singleplex run. After an initial activation step at 50°C for 2 min and a denaturing step at 95°C for 2 min, 40 cycles were performed as follows: 15 s at 95°C, 30 s at 55°C, and 60 s at 72°C. A melt curve was also performed to ensure a single qPCR product was formed, going from 50°C to 95°C at an increase of 0.5°C per cycle. The program was run on a 4-channel Q Real-Time PCR thermocycler (Quantabio, Beverly, MA) along with the Q-qPCR v1.0.1 software analysis program (Quantabio), which was used to determine the sample concentrations as compared to a standard curve. The efficiency of the PaV-LD major capsid protein primer set is 99.66% (Table 1).

### 2.4. Metagenome sequences for identification of cyanophage genomes

Two cell faction samples with high viral loads (ODNR4 on June 28, 2017, and ODNR1 on June 26, 2018) were chosen based on qPCR quantification of PaV-LD for metagenome sequencing. Samples were sequenced at the University of Michigan Advanced Genomics Core (Ann Arbor, MI) on a NovaSeq 6,000 sequencing system (Illumina, San Diego, CA). Once sequenced, FASTA files were imported into CLC Genomics Workbench v.20.0.2 software (Qiagen, Redwood City, CA) with the default quality settings following Steffen et al. (2017). Failed reads were discarded during import. Paired-end reads for both samples were trimmed for quality prior to being combined for assembly into contigs (minimum length of 2,000 bp) using the CLC Genomics Workbench *de novo* assembly function that also mapped reads back to the generated contigs. Contigs generated from ODNR 4 on June 28, 2017 were denoted with the data set number 140939 while contigs generated from ODNR1 on June 26, 2018 were denoted with the data set number 140938.

To supplement these two samples, metagenome generated contigs were obtained from a SPAdes analysis project (Ga0209229) from JGI Gold Biosample ID Gb0059903, a Sandusky Bay metagenome study dated June 11, 2013.

### 2.5. Identification of PaV-LD like cyanophage genes

To identify PaV-LD like cyanophage genes, the genomic sequence of PaV-LD (NC_016564) was obtained from NCBI and imported into CLC Genomics Workbench v.20.0.2 software (Qiagen, Redwood City, CA). Gene annotations were extracted using the Extract Annotated Regions 1.4 tool, which was used as a BLASTn database for metagenome generated contigs using standard parameters, except for an increased word size of 50. Positive BLASTn hits were filtered using a Lowest E-value of 0.00, Greatest identity % ≥ 85, and Greatest bit score of ≥1,500. The resulting hit sequences were exported to Geneious Prime (Biomatters Ltd., Auckland, NZ) version 2020.2.3 as a sequence list. Confirmation of relatedness was determined by mapping the contigs to the reference sequence PaV-LD (NC_016564) using Geneious mapper set to Dissolve contigs and re-assemble, Low Sensitivity, and Iterate up to 5 times. The rest of the parameters were kept standard. Regions of mapped coverage were extracted and re-aligned using MUSCLE 3.8.425 to determine percent identity between the viral genes of known function and the contigs. The DNA sequences were translated using the Translate tool and standard genetic code. Protein sequences were re-aligned using the Geneious Alignment tool with Global alignment and Blosum90 cost matrix selected.

### 2.6. Identification of novel *Planktothrix agardhii* cyanophage contigs

Metagenome generated contigs from 2017 and 2018, along with the downloaded contigs from 2013, were analyzed by BLASTn against Sandusky Bay *P. agardhii* isolate CRISPR spacer sequences as generated in McKindles et al. (2022). BLASTn parameters were modified to accommodate small sequences as follows: Match/Mismatch and Gap Costs were at Match 2, Mismatch 2, Existence 5, and Extension 2, Expectation value was set to 10.0, and Word size set to 25. Contig sequences with multiple hit regions were checked for full *P. agardhii* CRISPR-cas cassettes and were extracted if they contained novel spacer sequences. These new spacer sequences were added to the spacer sequence database generated previously and are described in Supplementary Table S2. All contigs were then re-analyzed by BLASTn against the newly generated full CRISPR spacer sequence database with the same BLASTn parameters as before. Any hit contigs with CRISPR-cas casettes and/or CRISPR spacer repeat sequence were removed and the remaining contig sequences (71 sequences) were exported as FASTA files for the detection of virus-associated sequences using VirSorter 1.0.3, with the “virome” option (Roux et al., 2015). Suspected prophages (no sequences) and contigs with no viral signatures (47 sequences) were then excluded from further analysis, leaving 24 contig sequences as possible viral sequences. A list of contigs that had CRISPR positive hits but did not pass the VirSorter viral signature analysis can be found in Supplementary Table S3. Several of these contigs were identified as viral homologues against PaV-LD but may not have contained the viral signatures that are searched for using the VirSorter database.

The 24 punitive viral contig sequences were imported in VIPtree (Nishimura et al., 2017) for the generation of a proteomic relatedness tree (generation of genomic similarity scores) and to annotate viral genes with tBLAST function. This tree was used to group similar sequences for in depth analysis of gene function, but given the short length of the viral fragments, may not accurately represent different viral sequences or families.

### 2.7. Metatranscriptome analysis from Sandusky Bay in 2015, 2018, and 2019

Extracted RNA was sent to Discovery Life Sciences (Huntsville, AL), where the samples were treated to reduce rRNA using TruSeq Stranded Total RNA with Ribo-Zero Plant kit (Illumina, San Diego, CA). RNA was sequenced on an Illumina HiSeq 2,500 platform with paired-end reads of 50 base pairs (2015) or 150 base pairs (2018, 2019).

The metatranscriptome reads were analyzed using the CLC Genomics Workbench v.20.0.2 software (Qiagen, Redwood City, CA). The raw reads were filtered through quality control where failed reads were removed using the CLC Toolbox, remaining reads were trimmed with an ambiguous base limit of 2 and automatic read-through adapter trimming. Reads shorter than 15 bp were discarded. The raw reads were mapped to whole punitive viral contigs generated above, the whole PaV-LD genome (NC_016564), and the whole genome of *Planktothrix agardhii* NIVA-CYA 126/8 (NZ_CM002803) using the RNA-Seq Analysis feature in the CLC Toolbox with a mismatch cost of 2, insertion cost of 3, deletion cost of 3, length fraction of 0.8, a similarity fraction of 0.8, and a maximum number of hits at 10.

### 2.8. Data availability

Raw read files from ODNR4 on June 28, 2017 (140939), and ODNR1 on June 26, 2018 (140938), were uploaded to the National Center for Biotechnology Information (NCBI) Sequence Read Archive (SRA) under Bioproject accession number PRJNA940836. The *Planktothrix* viral contigs assembled from the metagenome data sets (2017 and 2018) were deposited in the NCBI GenBank Database under accession numbers OQ674116 – OQ674125. Metatranscriptomes from 2015, 2018, and 2019 were also deposited in the NCBI SRA under Bioproject accession numbers PRJNA946791 (2015), PRJNA941812 (2018), and PRJNA945377 (2019).

## 3. Results

### 3.1. Monitoring of PaV-LD presence by quantitative PCR

**Figure 2:**
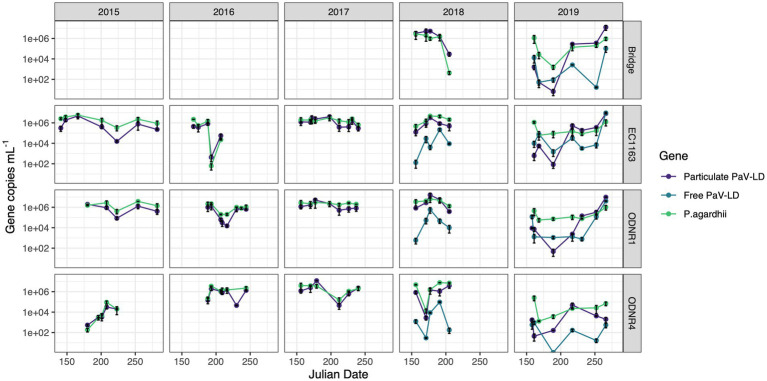
Quantitative PCR of PaV-LD like cyanophages and their host, *Planktothrix agardhii*, across five bloom-forming years in Sandusky Bay (Table 1). *Planktothrix agardhii* was quantified using the single copy housekeeping gene, *rpoC1*. PaV-LD like cyanophages were quantified using the major capsid protein gene sequence (PaVLD_ORF073R). Samples were particulate associated (0.22 μm filter) or free cyanophage (cation charged filter). Samples were analyzed as biological duplicates and standard deviation is indicated by the black bars.

The abundance of *Planktothrix agardhii* in Sandusky Bay was estimated using the single copy housekeeping gene, *rpoC1* (Figure 2). In 2015, *P. agardhii rpoC1* concentrations fluctuated between 186 and 5.76 × 10^6^ gene copies mL^−1^, with higher average concentrations occurring in the outer bay (EC1163 and ODNR1) compared to the inner bay (ODNR4). All three sites showed a decrease in numbers on August 11, 2015, which rebounded in both EC1163 and ODNR1. In 2016, *P. agardhii* concentrations ranged from 62 to 3.44 × 10^6^ gene copies mL^−1^. The low end of this range occurred on July 11, 2016, at site EC1163, where abundance of 1.49 × 10^6^ gene copies mL^−1^ dropped rapidly, recovering the next sample date to 2.25 × 10^4^ gene copies mL^−1^. While not as rapid, ODNR1 also shows a population dip on July 25 and August 3, 2016. 2017 was the most stable year for *P. agardhii* according to genetic quantification. Except for July 31, 2017, at site ODNR4 where the genetic concentration was the lowest for the year at 1.59 × 10^5^ gene copies mL^−1^, all *P. agardhii* concentrations were above 1.1 × 10^6^ gene copies mL^−1^. The last trip acquiring genetic information occurred on July 24, 2018, which was correlated with a population decrease at the Edison Bridge, EC1163, and ODNR1, while populations remained high in the inner bay at site ODNR4. ODNR4 in 2018 had a rapid decline in population early in the season, dropping from 4.73 × 10^6^ gene copies mL^−1^ to 1.4 × 10^4^ gene copies mL^−1^ before recovering to 1.58 × 10^6^ gene copies mL^−1^. These trends in qPCR estimation for 2018 were reflected in the *Planktothrix* biomass cell count data (Supplementary Figure S1). 2019, the final year of molecular quantification in Sandusky Bay as part of this study, was the most distinct. Gene copy numbers started off the season with some of the lowest values recorded at each site, recovering to more typical concentrations by August, and ending the season in September with continually high numbers. Overall, the highest genetic quantification of *P. agardhii* occurred multiple times in 2018 at sites EC1163, ODNR1, and ODNR4, while the lowest numbers occurred primarily at site ODNR 4 in 2015, 2018, and 2019.

Over this period, the presence of PaV-LD and local derivatives of this cyanophage were monitored by testing for the genetic sequence coding for one of the two major capsid proteins (Figure 2). In general, the genetic quantification of the capsid protein mirrored the trends of the host quantification. If the host population declined rapidly, the cyanophage genetic signature also declined. In 2015, the proportion of cyanophage genetic sequence compared to the host genetic sequence ranged from less than 5% (EC1163, August 11, 2015) to almost 3 × more prevalent than its host (June 29, 2015, at ODNR4). In 2016, both ODNR4 and ODNR1 measured lower viral population presence compared with its host, but EC1163 showed increased viral presence mid to late season (July 11, 2016, at 6.9 x more and July 25, 2016, at 2.5 x more). In 2017, cyanophage to host ratios were consistently between 0.23 and 3.29, further indicating that 2017 was a stable year. On July 28, 2018, at the Edison Bridge the first instance of a double-digit virus to host ratio occurred, which was 68 × more cyanophage than *P. agardhii*. Otherwise, ratios were more stable, where presence was measured at the lowest at 0.14 and up to 5.33 × more abundant. Finally, in 2019, some of the lowest concentrations of cyanophage genetic signatures were recorded in the early season, which made up only 0.02–1% of the host population concentrations. In mid-season, viral concentrations rebounded, matching the concentration of the host (approximately 1.3 to 3.6 x more virus than host) and ended the season at even higher ratios at the Edison Bridge (13.5 x), EC1163 (6.5 x), and ODNR1 (9.5 x). Given the similar patterns of the cyanophage-host abundance as outlined by this dataset, it becomes clear that these viruses are present and a prevalent component of cyanobacterial harmful algal bloom ecology.

In 2018 and 2019, extracellular PaV-LD quantification of select sites was added by concentrating free cyanophages onto a cation coated filter. In general, this quantification revealed that the free PaV-LD fraction was lower than the host associated fraction (Figure 2). The three sites sampled in 2018 showed a free viral fraction that made up approximately 3 ± 6% of the host associated viral fraction. On the other hand, 2019 was characterized by higher free cyanophage loads in the early season (average 11 times higher than host associated loads), followed by lower free viral percentages the rest of the year (20 ± 30%). Note that both free phage and host associated phage loads increase rapidly at the end of the season.

### 3.2. Identification of PaV-LD like cyanophage contigs

**Figure 3:**
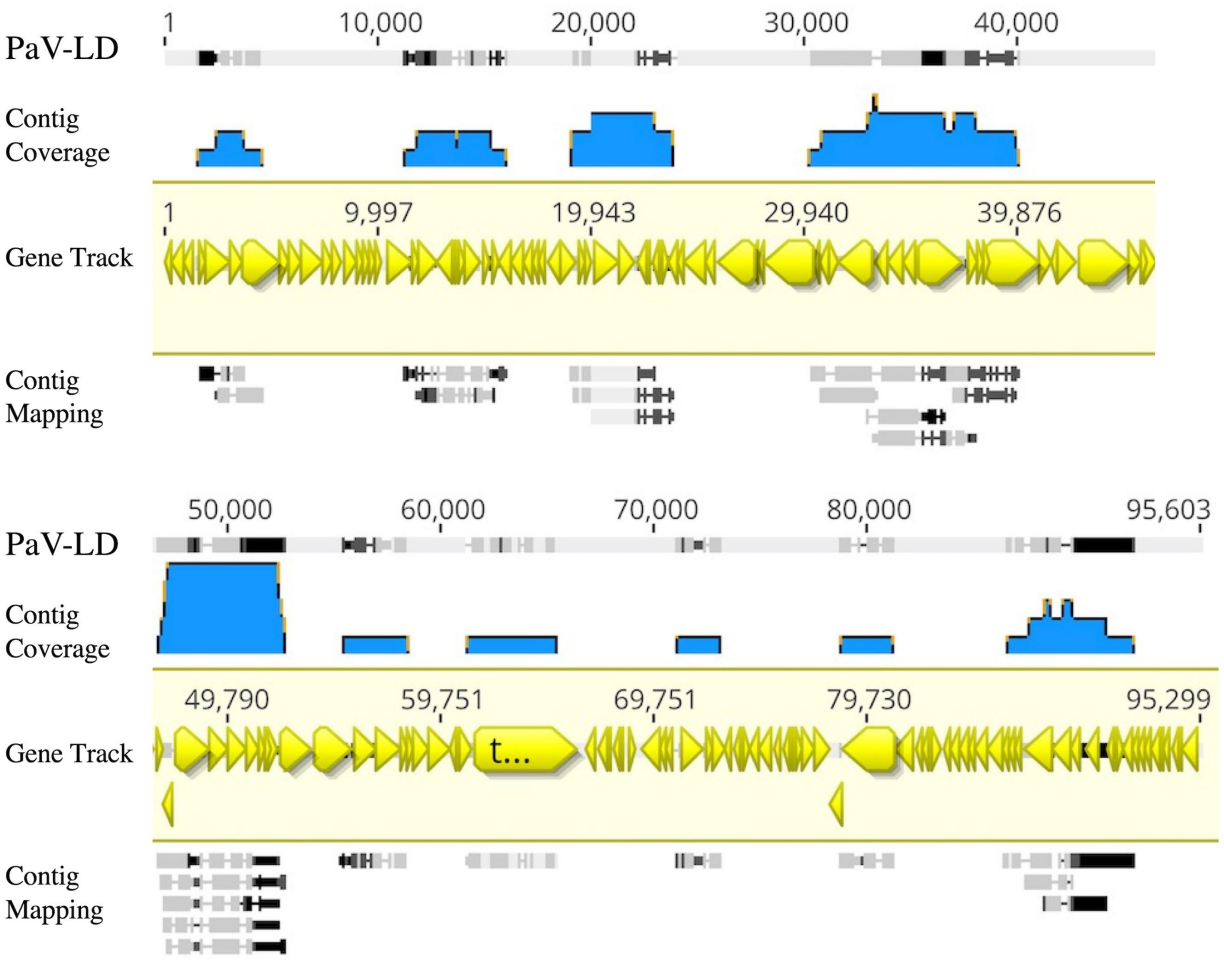
Contigs with CRISPR-cas identified viral signatures were aligned with the published genome of PaV-LD. 24 contigs were closely related (greatest identity % ≥ 85). White regions indicate 100% similar, light grey regions indicate greater than 80% similar, while both dark grey and black regions indicate discordant regions (insertions, deletions, and nucleotide substitutions). Reference sequence is highlighted in yellow and includes gene annotations. Coverage is noted using blue.

Metagenome generated contigs were mapped to the only known *Planktothrix agardhii* specific cyanophage, PaV-LD (NC_016564) to determine how related the PaV-LD-like cyanophages may be to the reference. From three metagenomic data sets, 24 contigs were closely related (greatest identity % ≥ 85) to genes from PaV-LD (Figure 3). These alignments can be classified as 100% similar (white regions, Figure 3), or greater than 80% similar (light grey, Figure 3), corresponding to the presence of conserved regions and genes. Discordant regions (Dark grey and black regions, Figure 3) are areas surrounding insertions, deletions, and nucleotide substitutions. These contigs cover several essential PaV-LD sequences, including both capsid proteins, the tail tape measure protein and a highly conserved region including a serine/threonine-protein phosphatase and a protein kinase (Table 2). In particular, the region encompassing both capsid proteins had the most coverage, where one capsid protein (ORF073R) was highly conserved both in nucleotide and amino acid identity, but the other (ORF071R) was only conserved in 3 out of 5 sequence contigs (Table 2).

**Table 2:** Known PaV-LD annotations that are covered by environmental metagenome generated contigs and their relatedness in nucleotide and amino acid identity.

PaV-LD gene annotation	PaV-LD ORF	Metagenome contig	Nucleotide identity	Amino acid identity
nblA	PaVLD_ORF022L	Ga0209229_10029639	97.58%	92.73%
		Ga0209229_10013247	98.18%	90.91%
Serine/threonine-protein phosphatase	PaVLD_ORF036R	140,939_contig_710	100%	100%
		140,938_contig_91	100%	100%
		Ga0209229_10010503	100%	100%
Protein kinase	PaVLD_ORF037R	140,939_contig_710	99.15%	99.26%
		140,938_contig_91	99.15%	99.26%
		Ga0209229_10010503	99.15%	99.26%
Terminase large subunit	PaVLD_ORF053L	Ga0209229_10001486	91.03%	78.13%
		Ga0209229_10026891	99.00%	97.20%
Portal protein	PaVLD_ORF057R	Ga0209229_10006839	99.35%	99.85%
		Ga0209229_10001486	91.23%	96.08%
Capsid protein	PaVLD_ORF071R	140,938_contig_201	97.05%	98.03%
		140,939_contig_132	97.05%	98.03%
		Ga0209229_10004734	96.98%	97.64%
		140,939_contig_131	89.58%	92.38%
		Ga0209229_10004679	86.15%	89.27%
Capsid protein	PaVLD_ORF073R	Ga0209229_10004734	98.64%	100%
		140,938_contig_201	98.85%	100%
		140,939_contig_132	98.74%	100%
		Ga0209229_10004679	92.77%	99.68%
Virion structural protein	PaVLD_ORF081R	140,938_contig_929	93.32%	85.68%
Tail tape measure protein	PaVLD_ORF088R	Ga0209229_10008835	96.89%	98.75%

### 3.3. Identification of novel *Planktothrix agardhii* cyanophage contigs

**Figure 4:**
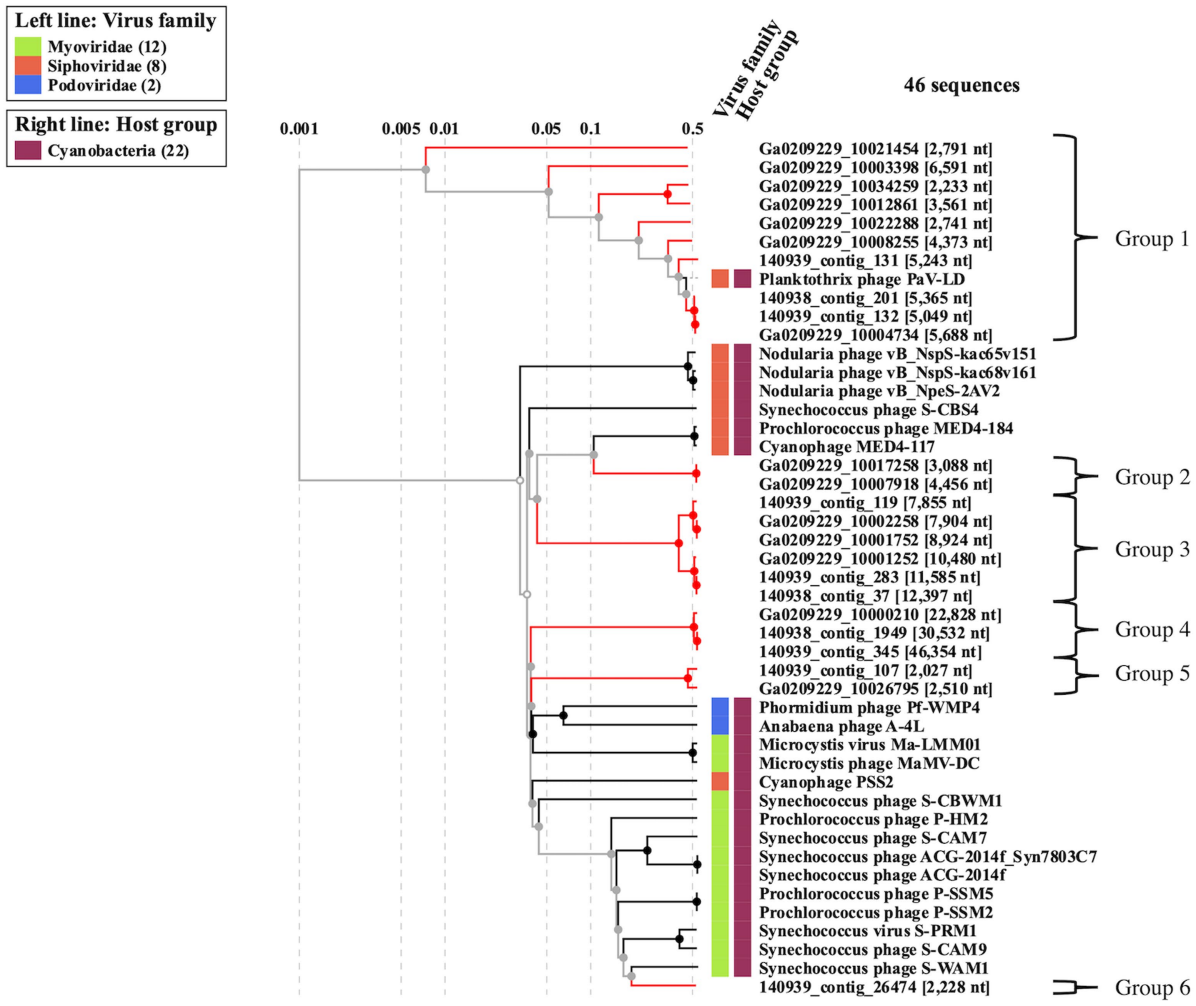
Proteomic viral tree (VIPtree) generated from metagenome generated contigs with positive CRISPR-cas spacer hits and viral signatures as identified by VirSorter. These 24 contigs can be placed into one of six proteomic groups, which may represent fragments of novel cyanophage genomes, or different segments of the same virus.

To better understand the cyanophage diversity present in Sandusky Bay, CRISPR-cas spacer sequences from previously sequenced *Planktothrix agardhii* genomes were used to identify metagenome contigs that were possible foreign genetic material. Additional curation using viral signatures yielded 24 contigs from 2013 (Ga0209229), 2017 (140939), and 2018 (140938), of lengths ranging from 2,027–24,459 bp (Table 3). The VirSorter Category for each contig is noted, where category 1 contigs are “most confident predictions” (significant enrichment in viral-like genes and at least one hallmark viral gene detected), category 2 contigs are “likely predictions” (either enrichment in viral-like genes or a viral hallmark gene is detected along with other metrics), and category 3 are “possible predictions” (neither a hallmark gene nor enrichment in viral-like genes but significant scores in other metrics) (Roux et al., 2015). VirSorter output includes a GenBank flat file which contains the number and location of genes for each contig, as well as predicted gene product. These annotations along with previous CRISPR spacer sequence alignments were used to determine the number of CRISPR spacer hits and the probable viral gene to which the spacers corresponded. Most of the spacer sequences hit for hypothetical proteins, some of which have a direct PaV-LD ORF associated with them. Others are related to phage clusters as defined by VirSorter. There were several contig sequences that had multiple spacers hit, and a few contigs that had spacers hit for more than one gene. Ga0209229_10034259 had the most CRISPR spacer hits at 19, which were at 4 different regions of the contig alongside each of the 4 genes. Ga0209229_10003398 had the second most hits at 11, which were focused on a single gene, indicating that this spacer sequence was common across 11 different *Planktothrix agardhii* isolates. A list of contigs that had CRISPR positive hits but did not pass the VirSorter viral signature analysis can be found in Supplementary Table S3. These 24 contigs were then used to generate a proteomic viral tree to understand their relationships to one another and to identify novel viral genetic signatures (Figure 4). This analysis identified 6 viral groupings, which mainly represent different gene functionalities. Note that these groupings are not indicative of all new viruses, and may represent different parts of the same virus.

**Table 3:** Characterization of environmental metagenome generated contig with viral signatures, including VirSorter confidence category, number of Sandusky Bay *Planktothrix* isolate CRISPR spacers hit to the contig, and the gene annotation of the spacer hit site.

Metagenome contig	VirSorter category	Proteomic viral group	Length (bp)	Number of genes	Genome average coverage	Number of CRISPR spacer hits
140,938_contig_201	1	1	5,399	7	3875.66	2
140,939_contig_131	1	1	5,273	9	1000.5	1
140,939_contig_132	1	1	5,113	8	5066.42	2
Ga0209229_10004734	1	1	5,688	9	N/A	2
Ga0209229_10022288	1	1	2,741	4	N/A	1
Ga0209229_10003398	2	1	6,591	4	N/A	11
Ga0209229_10008255	2	1	4,373	4	N/A	1
Ga0209229_10012861	2	1	3,561	9	N/A	10
Ga0209229_10021454	2	1	2,791	4	N/A	9
Ga0209229_10034259	2	1	2,233	3	N/A	19
Ga0209229_10007918	2	2	4,456	6	N/A	2
Ga0209229_10017258	2	2	3,088	6	N/A	2
Ga0209229_10026795	2	5	2,510	4	N/A	1
140,939_contig_107	2	5	2027	5	7256.96	1
140,939_contig_26474	2	6	2,251	6	8.85	2
140,938_contig_37	3	3	12,452	24	1240.89	2
140,939_contig_119	3	3	7,962	14	1669.29	1
140,939_contig_283	3	3	11,630	23	3013.87	2
Ga0209229_10001252	3	3	10,480	18	N/A	2
Ga0209229_10001752	3	3	8,924	15	N/A	1
Ga0209229_10002258	3	3	7,904	12	N/A	1
140,938_contig_1949	3	4	30,690	42	66.42	9
140,939_contig_345	3	4	46,459	74	194.87	9
Ga0209229_10000210	3	4	22,828	35	N/A	9

Genome coverage not available for Ga0209229 data set. Gene annotation for CRIPSR spacer hits can be found in Supplementary Table S4.

#### 3.3.1. Viral contig group 1 (PaV-LD)

Viral group 1 represents all the metagenome generated viral fragments that cluster on the same proteomic branch as the previously described *Planktothrix agardhii* cyanophage PaV-LD (Table 4). Some of the contigs are highly related to PaV-LD with genomic similarity (S_G_) scores above 0.95 or 95%, including Ga0209229_10004734, 140,939_contig_132, and 140,938_contig_201. Additionally, here are a few contigs that are partially related to PaV-LD with S_G_ scores above 0.75 or 75%, including 140,939_contig_131 and Ga0209229_10008255. Finally, there is a set of 4 contigs that are only loosely related to PaV-LD with S_G_ scores below 0.75 or 75%, including Ga0209229_10012861, Ga0209229_10034259, Ga0209229_10003398, and Ga0209229_10021454. All the contigs in this group have genes that were originally found in the sequencing of PaV-LD.

**Table 4:** Group 1 viral contigs and their genomic similarity to the reference PaV-LD and corresponding alignment to PaV-LD genes.

Group 1 Viral Contig	Genomic Similarity (SG) to PaV-LD	PaV-LD region hits
Ga0209229_10004734	0.9864	PaVLD_ORF070L to PaVLD_ORF077R
140,939_contig_132	0.993	PaVLD_ORF070L to PaVLD_ORF077R
140,938_contig_201	1	PaVLD_ORF070L to PaVLD_ORF077R
140,939_contig_131	0.8672	PaVLD_ORF070L to PaVLD_ORF077R
Ga0209229_10008255	0.7922	PaVLD_ORF078R and PaVLD_ORF079R
Ga0209229_10012861	0.466	PaVLD_ORF023R to PaVLD_ORF025R, PaVLD_ORF027R
Ga0209229_10034259	0.7238	PaVLD_ORF023R to PaVLD_ORF025R, PaVLD_ORF027R
Ga0209229_10003398	0.6411	PaVLD_ORF084R, PaVLD_ORF088R, PaVLD_ORF137L
Ga0209229_10021454	0.4704	PaVLD_ORF111R, PaVLD_ORF114L, PaVLD_ORF116R

**Figure 5:**
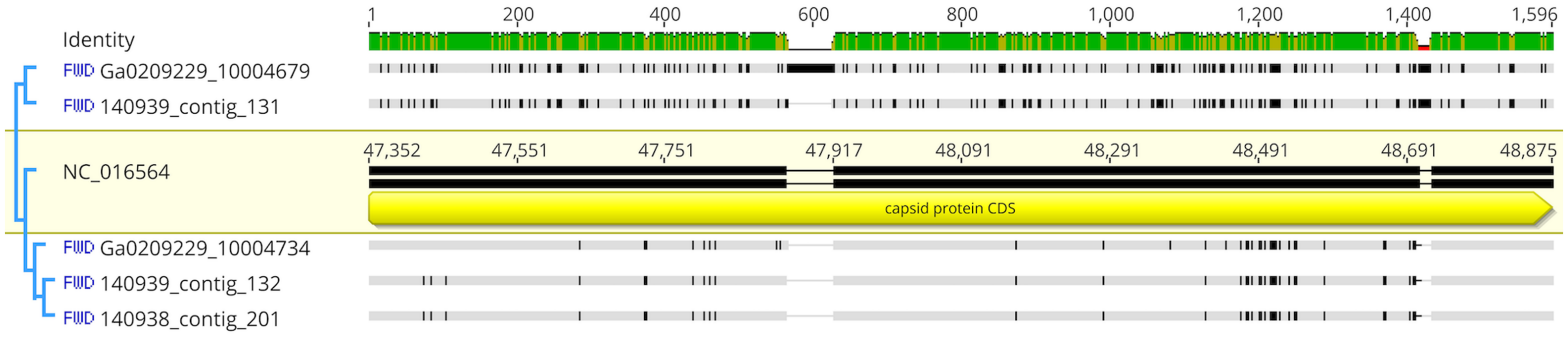
Nucleotide comparison of mapped contigs to PaVLD_ORF073R. Reference sequence is highlighted in yellow and includes gene annotations. Black segments in the non-highlighted sequences indicate points of difference, grey segments indicate similar regions. Identity is displayed at the top of the figure. Green sites indicate the same residue across all sequences while yellow sites have 30%–100% identity.

The 3 highly similar contigs (Ga0209229_10004734, 140,939_contig_132, and 140,938_contig_201) all encode for the same region of PaV-LD between 47 – 52 kb which includes the genes PaVLD_ORF070L to PaVLD_ORF077R. This region is mostly hypothetical proteins (6 out of 8 genes) but does include both capsid protein genes (PaVLD_ORF071R and PaVLD_ORF073R). Interestingly, this region of PaV-LD is also covered by the partially related contig 140,939_contig_131 and the contig Ga0209229_10004679, which was not picked up in the VirSorter analysis (Supplementary Table S3). As noted in previous analysis, the capsid protein PaVLD_ORF071R was covered by two distinct groups (Figure 5). The main group, including Ga0209229_10004734, 140,939_contig_132, and 140,938_contig_201, are highly similar to the reference amino acid sequence (~ 97% identity). The other group, including 140,939_contig_131 and Ga0209229_10004679, show a 95% amino acid sequence similarity to each other, but only an 88% amino acid sequence similarity to the reference. Interestingly, while closely related across years, there are some sequence differences between the 2013 data set (Ga0209229) and the 2018 and 2019 metagenomes, which may represent mutations acquired or lost over time.

The remaining contigs line up to other portions of PaV-LD (Table 4). These genes are primarily hypothetical proteins, with the exception of the tail tape measure protein PaVLD_ORF088R found in Ga0209229_10003398. Further, the contig gene hits do not have many conserved domains suggesting function, except for a relative of hypothetical protein PaVLD_ORF116R found in Ga0209229_10021454, which contains a KGG repeat domain indicating a possible function in stress response.

#### 3.3.2. Viral contig group 2

Viral group 2 consists of two approximately identical sequences, Ga0209229_10017258 and Ga0209229_10007918 (S_G_ = 1), which has a weak similarity to PaV-LD at S_G_ = 0.2282. These sequences encode for 5 genes: a DNA modification methylase (Phage_cluster_4886), an uncharacterized Tet_JBP domain-containing protein (Phage_cluster_6254), a nuclease with a partial alignment to PaVLD_ORF098R (Phage_cluster_4886), a hypothetical protein of unknown origin, and a hypothetical protein from *Planktothrix agardhii* (WP_235754195).

#### 3.3.3. Viral contig group 3

Viral group 3 consists of two different groups of three sequences each; Group 3A encompassing 140,939_contig_119, Ga0209229_10002258, and Ga0209229_10001752, while Group 3B includes 140,939_contig_283, 140,938_contig_27, and Ga0209229_10001252. Within subgroups, sequences are highly similar (S_G_ = 0.958 ± 0.026) and across the two subgroups, they are distantly related (S_G_ = 0.725 ± 0.009). Despite this lower level of proteomic similarity, the functionality of these sequences are similar across the clades (Supplementary Figure S2). Viral group 3 sequences generally encode for hypothetical proteins of unknown origin and hypothetical proteins from various cyanobacterial species (including *Nostoc* sp., *Trichocoleus* sp., and *Oscillatoria* sp.). Notably, these sequences also encode for a set of DNA-binding proteins including a HTH_XRE superfamily protein transcriptional regulator. The additional genes that are unique to subgroup B sequences include a NrdG superfamily protein related to the 7-carboxy-7-deazagunanine synthase QueE found in filamentous cyanobacteria, a PTPS (QueD superfamily) 6-pyruvoyl tetrahydropterin synthase found in some *Oscillatoriaceae*, and a GTP cyclohydrolase I FolE found in some *Nostacales*. These three genes are important in the conversion of GTP into 7-carboxy-7-deazaguanine (CDG) DNA modification.

#### 3.3.4. Viral contig group 4

Viral group 4 consists of three sequences, one contig from each year of metagenome data. These sequences are the largest from these datasets, with the smallest being Ga0209229_10000210 at 22.8 kbp and the largest being 140,939_contig_345 at 46.4 kbp. These three sequences are highly related (S_G_ ≥ 0.95) with a common core of ~30 genes, with the greatest difference between them being their size. While the majority of the open reading frames are hypothetical proteins of unknown origin, a few have functional annotations, including genes encoding an M23 family metallopeptidase (Phage_cluster_1627_PFAM-Peptidase_M23), a thymidylate synthase (Phage_cluster_1125_PFAM-Thymidylat_synt), a C39 family peptidase, a DNA polymerase III subunit beta (Phage_cluster_14685_PFAM-DNA_pol3_beta), a DUF5895 domain-containing protein, and a replicative DNA helicase (Phage_cluster_71_PFAM-DnaB_C). One additional functional annotation can be found in 140,938_contig_1949 and 140,939_contig_345 for a sugar kinase/Hsp70/actin family protein. As the longest contig, 140,939_contig_345 also has functional annotations for a DNA-cytosine methylase (DCM) superfamily protein (Phage_cluster_6007), a Nucleoside 2-deoxyribosyltransferase (RCL superfamily), a hypothetical protein which BLASTs to a hypothetical protein found in *Vibrio* phages and AAA family ATPases found in *Planktothrix sp*., a Von Willebrand factor type A (vWFA) superfamily protein found in some cyanobacteria, and three conserved domain of unknown function (DUF) containing proteins (DUF3846, DUF4926, and DUF1825).

#### 3.3.5. Viral contig group 5

Viral group 5 consists of only two short contig sequences, 120939_contig_107 and Ga0209229_10026795, which are quite similar (S_G_ = 0.8524). The similarity between these two sequences is primarily the result of a single large protein common between them, which BLASTp identifies as a hypothetical protein in the *Nostocales* but as a major capsid protein in other cyanobacteria species. Both also contain a Helix-hairpin-helix (HhH_5 superfamily) protein next to the hypothetical/capsid protein. The other open reading frames code for hypothetical proteins.

#### 3.3.6. Viral contig group 6

Viral group 6 consists of a single short contig, 140,939_contig_26474. It contains several hits from the genomic DNA of a freshwater uncultured Caudovirales phage, including a DnaB Replicative DNA helicase, a hypothetical protein, and a T4 gp41 helicase.

### 3.4. Metatranscriptome analysis from Sandusky Bay in 2015, 2018, and 2019

**Figure 6:**
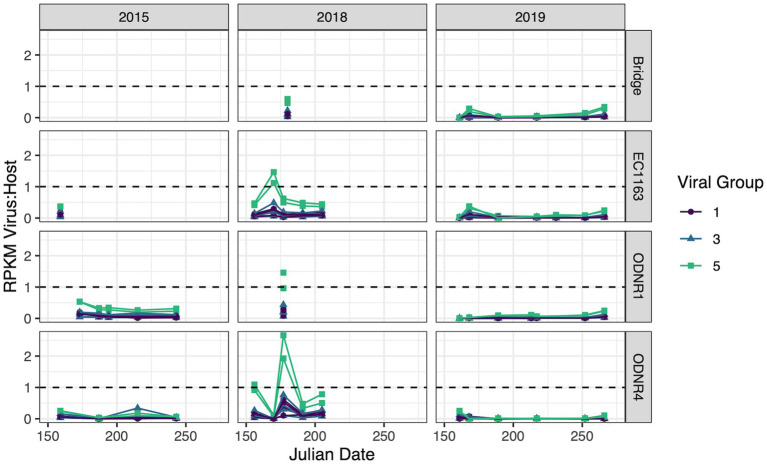
Transcripts of putative viral sequences categorized by group and normalized by whole genome expression of *Planktothrix agardhii*. Relative transcript abundance is presented as reads per kilobase of transcript per million mapped reads (RPKM). Viral groups with RPKM consistently less than 10% of the host RPKM were removed. Dashed line represents a viral to host transcript ratio of 1, where higher values indicate an increased likelihood of widespread active infection.

Samples for metatranscriptome analysis were collected in 2015, 2018, and 2019 during *Planktothrix*-dominated blooms in Sandusky Bay. These data sets were then used to analyze the transcriptomic activity of the cyanophages and foreign DNA identified in this work. All contigs had reads associated with them, but contigs that displayed consistently low Reads Per Kilobase Million (RPKM) compared to *Planktothrix agardhii*, as determined by a viral RPKM consistently less than 10% of the host RPKM, were removed. Only viral groups 1, 3, and 5 had contigs that displayed elevated gene expression (Figure 6).

Viral group 1 was driven by Ga0209229_10004734, 140,939_contig_132, and 140,938_contig_201, the contigs encoding for highly similar capsid proteins as PaV-LD (~97% amino acid similarity). These viruses collectively had peak gene expression on June 26, 2018 with a virus:host RPKM ratio of 0.504 ± 0.097 at ODNR4. They were also seen at elevated expression levels at all three sites in 2015 and 2018 (greater than 0.1, less than the peak of 0.504), but exhibited low expression in 2019. Only one other viral group 1 contig was found to have expression values above the threshold; 140,939_contig_131 within the secondary group encoding the variant major capsid protein of PaV-LD (~88% amino acid similarity). Like the other PaV-LD contigs, 140,939_contig_131 showed elevated expression levels on June 26, 2018 (ODNR4) at a virus:host RPKM ratio of 0.102, but peaked on June 8, 2015 (EC1163) at a virus:host RPKM ratio of 0.194.

For viral group 3 contigs, both subgroups saw expression levels above the threshold. Viral group 3A was represented by Ga0209229_10002258 and 140,939_contig_119. Ga0209229_10002258 had higher expression levels more frequently, with virus:host RPKM ratios over 0.1 across 20 site and date combinations. It was most prevalent in June 2018 in the outer bay of Sandusky (ODNR1 and EC1163) at a ratio of 0.46 ± 0.025 and found across all 3 years above the threshold. 140,939_contig_119 had less frequent elevated expression levels, with only 7 site/date combinations above the threshold, limited to the year 2018. Similar to the viral group 1 sequences, this contig peaked on June 26, 2018, at a ratio of 0.426. Viral group 3B was only represented by a single contig: Ga0209229_10001252. Ga0209229_10001252 displayed a similar trend in expression to viral group 3, clade 1 representative Ga0209229_10002258 in that it had quantifiable expression levels in 20 site/date combinations and spanned multiple sites across all 3 years. But unlike the 3A sequence, Ga0209229_10001252 peaked in August 2015, at a RPKM ratio of 0.34. The next highest expression level for this group occurred in June and July of 2018, averaging 0.27 ± 0.033 at sites ODNR4 and EC1163.

The most viral transcript expression was reported from both viral group 5 contigs (Figure 6). In June 2018, across all three sites, these two viral contigs had an average virus:host RPKM ratio of 1.45 ± 0.55, indicating that these genes were expressed approximately 1.5x greater than the host housekeeping gene of the sampled population. These sequences also had elevated expression levels on June 22, 2015 (ODNR) at a ratio of 0.532 ± 0.002, and throughout July 2018 at an average ratio of 0.498 ± 0.122. Interestingly, these sequences are the only ones that show considerable expression levels in 2019, peaking on July 17^th^ (EC1163) at a ratio of 0.344 ± 0.03. In total, these two contigs accounted for 57 site and date combinations where their transcript expression levels were above the threshold.

## 4. Discussion

Using metagenome and metatranscriptome data sets from the environment combined with *Planktothrix agardhii* isolate genomes from the same region, early work is presented here that identifies possible cyanophage and other foreign DNA associated with *P. agardhii*-dominant cHABs in Sandusky Bay, Lake Erie. Previous work identified the CRISPR-cas systems of Sandusky Bay *P. agardhii* isolates, noting that two of these gene cassettes were common across all isolates and two previously published reference sequences from other geographical regions (McKindles et al., 2022). That same study found that only 14.9% of the CRIPSR spacer sequences from the isolates and reference sequences could be attributed to PaV-LD, indicating that there was a hidden diversity of cyanophages and foreign DNA elements to be uncovered. Using methodology of Morimoto et al. (2019) for the elucidation of novel *Microcystis* cyanophages, some of this hidden diversity was elucidated. In brief, a non-redundant CRISPR-cas spacer sequence was created as a query against punitive viral contigs identified using the program VirSorter from environmental metagenomic samples as a method to identify host-virus interactions in the absence of laboratory isolates.

A first analysis using environmental metagenome data sets was to check for local variants of the already characterized *Planktothrix agardhii* cyanophage PaV-LD (Gao et al., 2009, 2012a). Several contigs were able to map to PaV-LD at a greater than 80% sequence similarity (Figure 3), indicating that PaV-LD like cyanophages can be present across geographical regions with region specific variations. While this is not the first study to identify PaV-LD signatures using ‘omics data sets, it is the first to confirm the presence of PaV-LD like cyanophages outside of Lake Donghu, China (Gao et al., 2012a). Watkins et al. (2015) discovered PaV-LD signatures in Lake Michigan, another of the Laurentian Great Lakes, but noted that while a BLAST search identified the hits as PaV-LD, they were likely non-species or non-virus specific ABC-transporter homologs. Similarly, Potapov et al. (2022) identified the same ABC-transporter identified as PaV-LD in Lake Baikal from metatranscriptome data but disregarded the presence of PaV-LD as the host cyanobacterium (*Planktothrix agardhii*) was not known to be found in the lake. The analysis presented here was also able to identify partial sequences aligned with this ABC-transporter, but identified it only as part of a larger contig that covered the PaV-LD region inclusive of ORF033R – ORF037R, lending multiple gene support to the contig’s identification (Figure 3). Besides this region, contigs inclusive of PaV-LD specific proteins such as the terminase large subunit, the portal protein, both major capsid proteins, and the tail-tape measure protein were identified (Table 2). In particular, the terminase large subunit (TerL) is so specific, it has been used in other studies as a phylogenetic marker for viral relatedness (Jin et al., 2020; Lin et al., 2020). This region was mapped by two related but distinct contigs (Table 2), perhaps denoting the presence of viral evolutionary diversity in response to host diversity. This trend of two related but distinct contigs was also noted in the major capsid proteins (PaV-LD ORF071R and ORF073R; Table 2). PaV-LD has a capsid size of 76 ± 6 nm and is genetically distinct when compared to the major capsid proteins of other cyanophage viral families (Gao et al., 2009, 2012a). Given the conserved regions of these genes between PaV-LD and the local contigs, isolates within this group may have similar characteristics. But the level of conservation was not the same across all contigs (Figure 5), again leading to the hypothesis that there may be evolutionarily related cyanophages present in Sandusky Bay that may be specific to different ecotypes of *Planktothrix agardhii* as a response to host diversity. While some freshwater cyanophage capsid structures have been elucidated by cryoelectron microscopy (Jin et al., 2019; Cui et al., 2021), without isolation, capsid structure and assembly of Sandusky Bay PaV-LD like cyanophages is out of reach.

Besides PaV-LD like cyanophages, the host CRISPR-cas spacer sequences were used to identify novel viral signatures with the idea of enhancing understanding of cyanophage diversity as part of an ecosystem dominated by a freshwater harmful algal bloom. Through this analysis, 5 proteomic groups were identified with potentially novel viral signatures (Figure 4). It should be noted that given the short sequences identified in many of the groups, it is unclear that each proteomic group represents a different viral family, but that multiple groups may be fragments of the same novel family or that the group identifies common viral genes found across multiple viral families. Despite this caveat, each of the 5 groups presented as part of this study have gene annotations that support the idea that cyanophage diversity is quite large despite the limited number of freshwater cyanophage isolates to date. Group 2 cyanophage contigs (Figure 4) contain a nuclease and DNA methylase genes which are involved in phage DNA replication and can be used to counter bacterial defense systems. Both gene functions can be found in PaV-LD (Gao et al., 2012a) and represent some of the most common phage orthologous groups (POGs; Kristensen et al., 2013). Group 3 cyanophage contigs had a HTH_XRE superfamily gene which may be functionally related to Cro/CI repressors which act as regulators of the lytic and lysogenic life cycle switch, as found in other systems (Wood et al., 1990; Basso et al., 2020; Long et al., 2022). Group 3B cyanophage contigs were longer than group 3A contigs (Figure 4) and had some additional genes that were identified as part of a secondary metabolite cassette. This cassette is important in the conversion of GTP into 7-carboxy-7-deazaguanine (CDG) for the biosynthesis of all 7-deazapurine-containing compounds (also known as pyrrolopyrimidine-containing compounds; McCarty and Bandarian, 2012). While the isolates of *Planktothrix agardhii* from Sandusky Bay have a 6-carboxytetrahydropterin synthase and several different copies of GTP cyclohydrolase I (McKindles et al., 2022), they are not the same as those found in the viral contig, nor does *P. agardhii* have the flanking gene, the 7-carboxy-7-deazagunanine synthase QueE. In *P. agardhii*, these genes are part of a pathway to produce Queuosine (Q), a hypermodified 7-deazaguanosine nucleoside located in the anticodon wobble position of four amino acid-specific tRNAs (Harada and Nishimura, 1972; Reader et al., 2004). In some viruses, 7-deazaguanosine is used during DNA modification to protect against host restriction enzymes (Hutinet et al., 2019). Next, group 4 viral contig sequences were the longest contigs generated from these environmental datasets (Figure 4). This group contains three contigs of variable size, but a core of ~30 genes between them. They have a M23 metallopeptidase gene, like the one encoded in PaV-LD ORF123, which has been shown to have bacteriostatic effects, including growth inhibition and membrane damage (Meng et al., 2022). Additionally, these contigs have an annotation for a thymidylate synthase, which can be found in both freshwater and marine double-stranded DNA viruses (Graziani et al., 2004; Yoshida T. et al., 2008; Huang et al., 2012; Zhang et al., 2020). Thymidylate synthase can be used by phages for catalyzing cyanophage-encoded nucleotide biosynthesis and scavenging of host nucleotides (Graziani et al., 2004; Thompson et al., 2011; Huang et al., 2015). Other genes of note in this groups include a DNA polymerase, helicase, and other enzymes important in the replication of viral genetic material. Despite being the most active viral group (Figure 6), group 5 viral contigs possess only two annotations of note involving a hypothetical capsid protein adjacent to a Helix-hairpin-helix family protein, which is likely related to non-specific DNA binding (Shao and Grishin, 2000). Finally, group 6 consists of only a single contig (Figure 4) which contains genes for a DnaB replicative DNA helicase and a T4 gp41 helicase, both of which are necessary to ensure proper regulation of cyanophage DNA replication initiation and which have been found in Nostoc and marine cyanophages (Dassa et al., 2009; Sullivan et al., 2010; Chénard et al., 2016).

In tandem with the sampling schedule for ‘omics datasets, PaV-LD-like cyanophages in the environment were quantified using quantitative PCR methodology. This is the first multi-year data set in which the presence of a freshwater cyanophage was monitored and quantified in the environment. Note that this analysis targets the host-and particle-associated viral fraction, which can include phage particles attached to the host, phage undergoing active lytic infection, and lysogenic and/or integrated cyanophages. This analysis also targets one specific gene, the major capsid protein, using a set of primers designed from the previously published reference sequence (Gao et al., 2012a). Quantification showed that except for a few dates, the genomic copy numbers of PaV-LD-like cyanophages, as determined by the major capsid protein PaV-LD ORF073R, mirror the concentrations of the host genome (Figure 2). Coincident with changes in host abundance, changes in the viral concentration were typically observed. This relationship seems to denote a constant presence of cyanophage which may not affect the duration or intensity of the bloom. Single year quantification of *Microcystis* cyanophages in Singapore showed similar relationships between host cell concentrations and host-associated viral loads between mid-July and early-August, noting that despite the high host-associated load, there was no corresponding high free phage load that would indicate active lytic infections (Zhang et al., 2022). Similarly, despite being a log or two less abundant than its host, *Microcystis*, Ma-LMM01 concentrations in the host cell fraction mirrored host concentrations in a Japanese pond, and high concentrations in the host fraction did not necessarily signal high concentrations in the free phage fraction (Kimura et al., 2012). Both studies suggested that the higher proportion of host-associated viral loads was due to rapid diffusion of free phage and the likelihood that viral progeny may be trapped in host colonies and mucilage. Alternatively, lysogenic genes have been found during *Microcystis* dominated blooms, and the shift between these genes and lytic infections was tied to environmental cues (Stough et al., 2017). Indeed, Ma-LMM01-like cyanophages may utilize lysogeny to replicate during bloom formation in a host that is rapidly growing and can persist at high densities. Given that the viral loads as part of the host-associated fraction in this study were similar to the host concentration, transcriptomics data were assessed to check the gene expression of the viruses. Despite the elevated qPCR quantification of the viral genomes, the transcription levels of these viral genes (Group 1) were not very high (Figure 6). We further analyze this trend with the 2018 and 2019 free phage fraction, noting that in 2018, free cyanophage was less than 10% of the host associated fraction (Figure 2). This may indicate that the viruses could be part of abortive infections, or even undergoing lysogeny. In 2019, free cyanophage concentrations were much higher, particularly in the early season, which may be related to the lower host concentrations found in the bay.

Whereas the transcriptional activity of PaV-LD-like cyanophages was low on most sample dates, the environmental transcriptomic analysis revealed elevated gene expression by group 5 viral contig genes relative to the host housekeeping gene rpoC1, perhaps capturing a viral event occurring throughout Sandusky Bay. Elevated transcriptional activity was found at EC1163 and ODNR4, two sites that are 25 km apart and which represent extreme ends of the embayment (Figure 6). As discussed above, group 5 has an annotation for a hypothetical major capsid protein not seen in any characterized cyanophage before, but since the CRISPR-cas spacer hits for this exact gene (Table 2), it is likely that this analysis has identified a novel *Planktothrix* cyanophage. Transcriptomics have been utilized to identify viral events before, which particularly focus on the transcript levels of capsid proteins as representative of late action viral genes (virion morphogenesis). The expression of late genes in a lab infection of Ma-LMM01 increased gradually until reaching a peak 6 h later, leading to host lysis (Morimoto et al., 2018). Another study noted that capsid assembly genes were diurnal and typically expressed after dark, which suggests that synchronized lysis of the host occurs during the night (Chen and Zeng, 2020). Unfortunately, due to low sample frequency, many lysis events can be missed, and the nuances of infection dynamics are lost.

While data are presented here with the goal of better understanding the relationship between freshwater cyanophages and their filamentous hosts, there are still many areas that need further research. It has been suggested that cyanophage infections can influence the cyanobacterial composition within a cHAB (Yoshida M. et al., 2008), so future work will examine more in-depth the transcriptomic data to determine if there was a *Planktothrix* shift following the proposed 2018 lysis event. Further, the proposed new viral genetic signatures could be quantified using historical DNA samples from the bay and better resolution of the viral sequences can occur once more metagenomic data sets are obtained. Finally, more research is needed into the triggers that promote cyanophages to become lytic to better inform water treatment plants when a mass lysis event will occur in reservoirs and waterbodies where these cHABs occur.

In conclusion, this study has found that PaV-LD-like cyanophages are a constant presence in *Planktothrix*-dominated cHABs but may not be regularly undergoing lytic infections. It also identified several new viral signatures that may be used to identify novel *Planktothrix*-specific cyanophages in other metagenomic data sets.

## Data availability statement

The datasets presented in this study can be found in online repositories. The names of the repository/repositories and accession number(s) can be found in the article/Supplementary material.

## Author contributions

KM: conceptualization and writing – original draft preparation. KM and MN: methodology. KM, MM, and MN: formal analysis and investigation. KM, MM, MN, RM, and GB: writing – review and editing. RM and GB: funding acquisition and resources. KM, RM, and GB: supervision. All authors contributed to the article and approved the submitted version.

## Funding

This work was supported by funding from the Ohio Department of Natural Resources (GB) and National Institutes of Health (1P01ES028939-01) and National Science Foundation (OCE-1840715) awards to the Bowling Green State University Great Lakes Center for Fresh Waters and Human Health (GB, RM). This work was also supported by funding from the Natural Sciences and Engineering Research Council of Canada, grant RGPN-2019-03943 (RM).

## Conflict of interest

The authors declare that the research was conducted in the absence of any commercial or financial relationships that could be construed as a potential conflict of interest.

## Publisher’s note

All claims expressed in this article are solely those of the authors and do not necessarily represent those of their affiliated organizations, or those of the publisher, the editors and the reviewers. Any product that may be evaluated in this article, or claim that may be made by its manufacturer, is not guaranteed or endorsed by the publisher.
