# Supplementary material for: Multi-year molecular quantification and ‘omics analysis of Planktothrix-specific cyanophage sequences from Sandusky Bay, Lake Erie
Source: Front Microbiol. 2023 Jun 29;14:1199641. doi: 10.3389/fmicb.2023.1199641 (PMC10343443; doi:10.3389/fmicb.2023.1199641)
Supplement: Supplementary file 1 [file Data_Sheet_1.docx]

Supplementary Material

Multi-year molecular quantification and 'omics analysis of *Planktothrix*-specific cyanophage sequences from Sandusky Bay, Lake Erie

Katelyn McKindles*^1,2,3^, Makayla Manes^4^, Michelle Neudeck^3^, R. Michael McKay^2,3^, George S. Bullerjahn^3^

*** Correspondence:** Corresponding Author: kmckindl@umich.edu

# Supplementary Figures and Tables

## Supplementary Figures


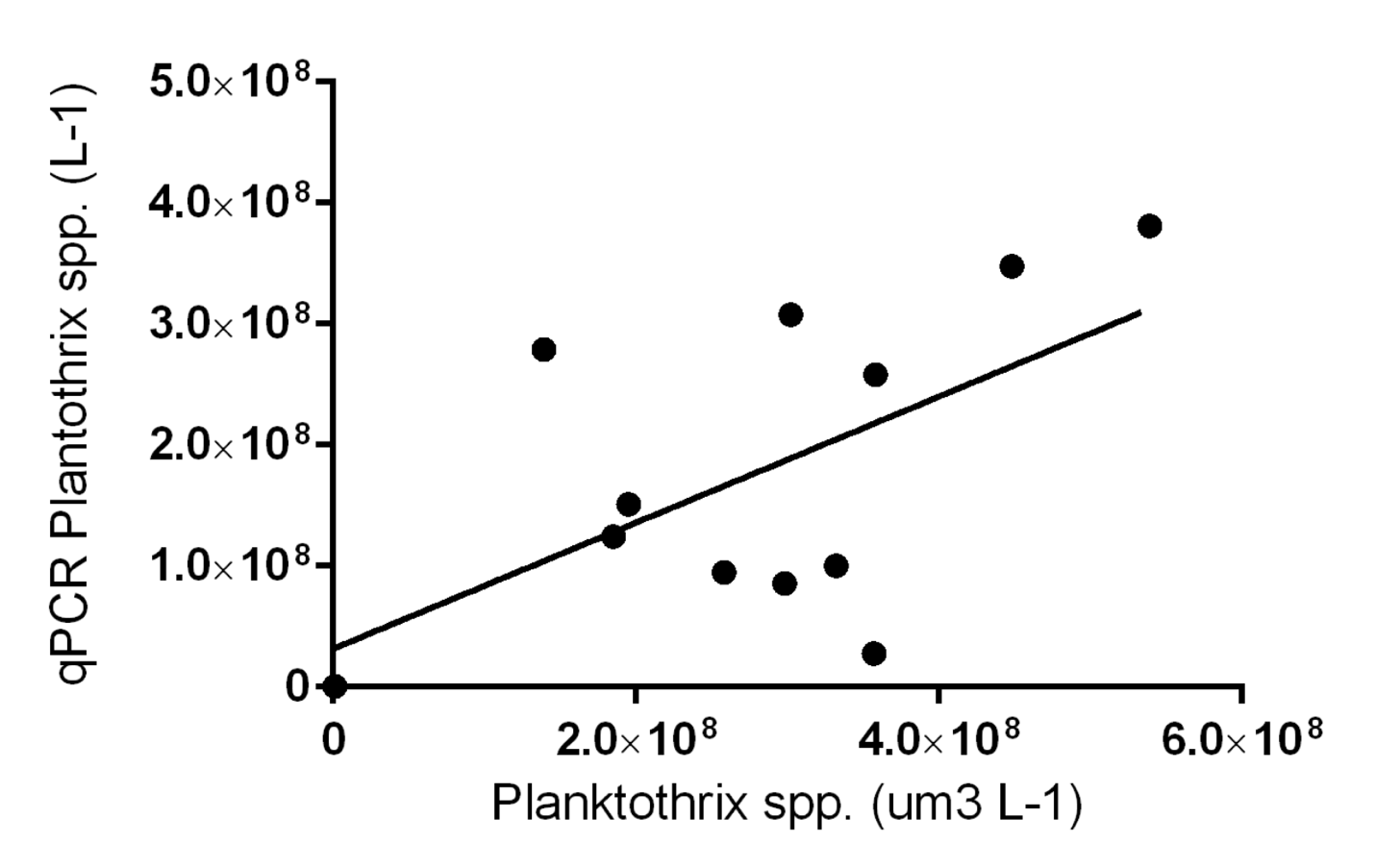


**Supplementary Figure 1.** Correlation between qPCR *Planktothrix agardhii* gene copies L^-1^ (rpoC1) and biomass calculations from microscope counts in µm^3^ L^-1^. Linear regression slope 0.5211 ± 0.2323 and has an r^2^ value of 0.3348. Comparison of data occurred from the 2018 data set at sites ODNR4 and EC1163 where biomass data was readily available (Bullerjahn and McKay, 2020).


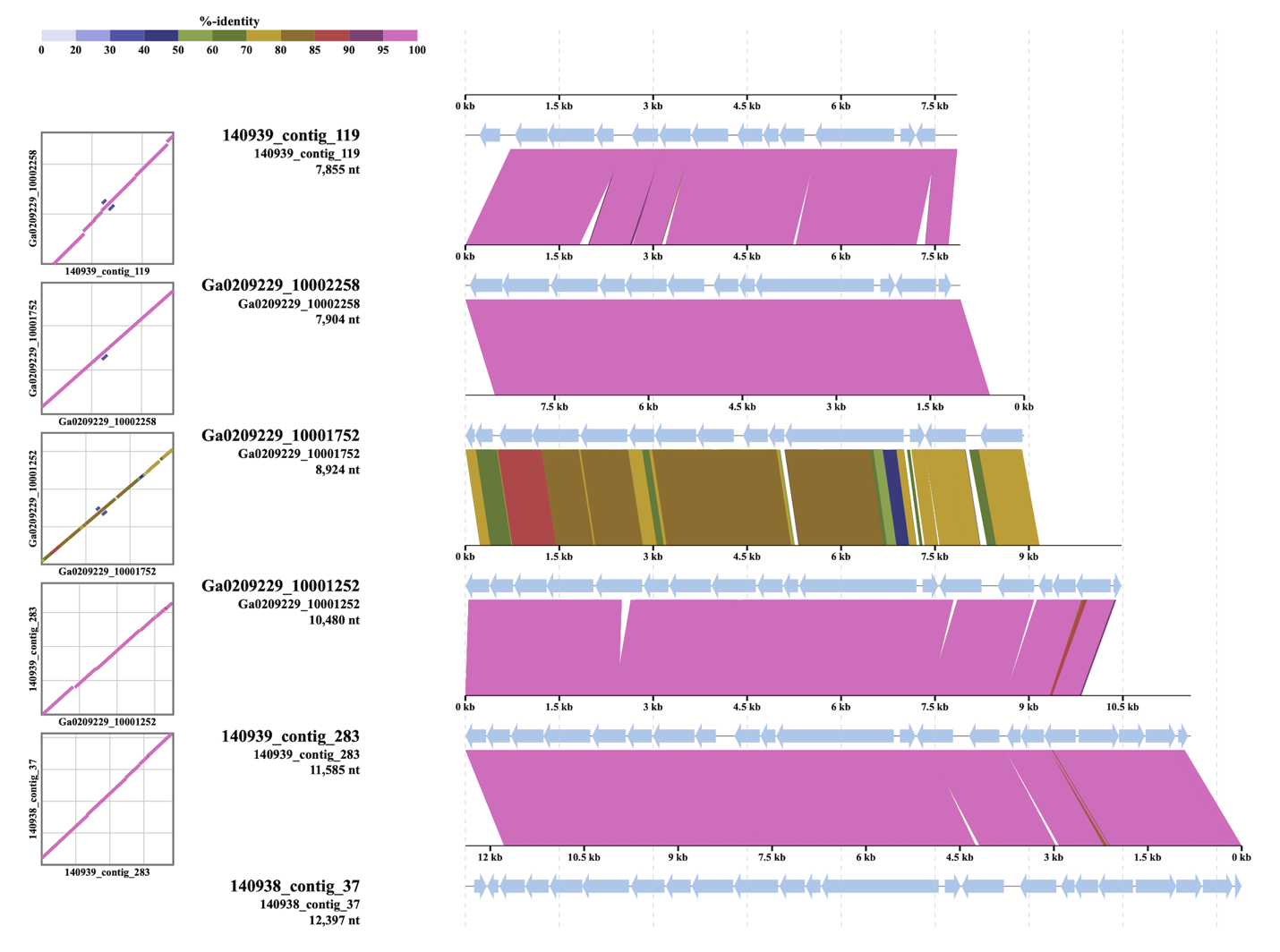


**Supplementary Figure 2.** Proteomic similarity between suspected viral signatures within *Planktothrix* cyanophage group 3. The two clades represent similar functionality but not genetic similarity.

## Supplementary Tables

**Supplementary Table 1.** G-block standard sequences for *Planktothrix agardhii* and PaV-LD.

| Target Gene | Standard Sequence | Standard curve range (copies μL ^-1^) |
| --- | --- | --- |
| *Planktothrix agardhii* rpoC | ATGTGGTGTTAAATCCAGGTAACTATGACGGCCTATCCTATAAACAGTTATTAACCGAAGATACTTGGTTAGAAATTGAAGACCAAATTTATAGTGAAGATTCCACCTTAACCGGAATTGAAGTGGGAATTGGAGCCGAAGCCATTTCCCGTTTGCTCGAAGATATTCCCTTAGAAGAAGAAGCCGAAAGATTACGGGAAGAAATTGCCGTTGCTAAGGGACAAAAACGCGCCAAA | 3.866 × 10^9^–38.66 |
| PaV-LD major capsid (ORF073R) | GGATGATATCGCGGCTGTTAGTCGGATGGGCGAGAGTATTGAAGAAGACAAAATCTGGACAATTCAAGAAGCCGCACAACAAAAAATCAATCAGTTGGTGGCAAACGGGGATTTAGAAACCGGAATGCCCGGTTTCTTGAATCATCCCCAAGCACTGCGATCTTATGCTCCCTTCCCTTTGAACGGGTCAGCAACATCACAGCAAAAACTGAGTGTATTAAATGATTGTGTTAATGCTCCGACTCGGCTGACCAATAACCGAGAGAAGCCGGACACATTGTTGATGGATTCGGAAACTTACGAACACCTCTCCTCGGATATTATTCAAATCGGCACATCCGCATTGGATCGTACGGTGTTAGAGCATTTCTTAAAAGTCAACCCCAATATCAAAGAAGTCGGCGTTGTGTCCGAGATGGCTCCTGACTATTTAGAGTCGATTGGTTTAGCTCCCACCCGATTTATTCAGGCTTT | 2.056 × 10^9^ – 20.56 |

**Supplementary Table 2.** Novel CRISPR-cas spacer sequences from JGI data set Ga0209229.

| >spacer1 | TTCTAAGCTCTACTGTCGCGTTATACGTTCCCACA |
| --- | --- |
| >spacer2 | TTTCAACATTCAAAAAGGGGGCGGCATGAAAAGAGA |
| >spacer3 | AACAAGCTGGTCAAATTAAAAACCAAGTCATCAAAAAACCAGA |
| >spacer4 | GAGGGAAAACCCAAGTTTCCTTCCCCCACTACCATTGCCTA |
| >spacer5 | ATAACAACAGAAAGATGAGCAGACAAAAAGGCTACGCGATTTGGGA |
| >spacer6 | AACAATGGAAGAATTTAACACTTTAATTAATTCAATTCTTGGAG |
| >spacer7 | ACCGCCTTAGAAGTGTTAGCAAACCCTGAGTTATTCGACG |
| >spacer8 | AAAAGAGGTATTTGATACATTCAAATTGCCTCGATACGT |
| >spacer9 | ACTAGAACTAGACTTAGATTTCCTCGTGTGGGATCTAGAC |
| >spacer10 | AGGATAACTGCCTCAATCAAGATACTCAGACAAGTGGGT |
| >spacer11 | TCCATAGGTGAAATTGTCCCAGGACAGGACTTGAGCCACGATTT |
| >spacer12 | TCATTAACGCCGCCGATTCATTGGGACGGGATGATTTAAT |
| >spacer13 | TTTTAAATCATCATCCCCTCGTCGCAAGGAGTCTTCTGACGG |
| >spacer14 | CATCTTGTACAACAAAGACAAGGTTTTTCAGTTGTTAAAAAC |
| >spacer15 | AATCACAATCGACACAATCTTAATGACCTACCC |
| >spacer16 | TACCTTTTATAAATAGAAACCTTGTGAGTTAATCAC |
| >spacer17 | TTTTAGCCTTAAATTGAGAACCTAAAAACGCATTACT |
| >spacer18 | TCTCGCCCATCCGACTAACAGCCGCGATATCATCCTCAGAGACGCTATAG |
| >spacer19 | ATTATGTCAGGAACTCAACCCCATACAAAGGCGAGATTTACC |
| >spacer20 | TTCCGATCCGTTTCTGACTTGATTGTATTCACTATCTTTTGGGG |
| >spacer21 | CGGACAGGGAACTGTCACAAAAGCTTTCGGAACGAAGAATCCGCTG |
| >spacer22 | TGCTCTACAAATGTAGAGCGATAGAGTAGAAAACCACCAA |
| >spacer23 | GGATAATGAATTGATTATTTATCGTCATCTGTATGAAGAAGGA |
| >spacer24 | TTCTACTAATACTTATCGTATGAGTGTTGAACTTAAAGTCTCTAACCC |
| >spacer25 | TGCCCATCCGTTGCAGGAGGGATTCTTTATCAGGGTCAACCA |
| >spacer26 | GTAGGTTTTGGAGTTGCTGCAGGGCGTTTTATT |
| >spacer27 | TGTATACCCCAACAGAACCATTAACTCCCGCGCCATCCAA |
| >spacer28 | AGTGTGATTAAAACTTTTTTATTAAGCATTCCTAACCGCTTTACATT |
| >spacer29 | TCCGTTACAGAATATTCCGTTTCTTTCCCATAGCCCGTTTCATA |

**Supplementary Table 3.** List of contigs that had CRISPR positive hits but did not pass the VirSorter viral signature analysis.

TableS3PlkVirus.xlsx

**Supplementary Table 4.** Blast annotation of CRISPR spacer hit site on suspected viral contig.

| Metagenome contig | Annotation where spacer hits |
| --- | --- |
| 140938_contig_201 | ORF4: PFam_DUF2184  ORF7: Hypothetical protein [PaV-LD] |
| 140939_contig_131 | ORF1: Phage cluster 1-r 1 |
| 140939_contig_132 | ORF1: PFam_DUF4054  ORF4: PFam_DUF2184 |
| Ga0209229_10004734 | ORF1: Hypothetical protein [PaV-LD]  ORF5: PFam_DUF2184 |
| Ga0209229_10022288 | ORF3: Phage cluster 5-r 1 (virion structural protein) |
| 140939_contig_107 | ORF4: Phage cluster 889 |
| 140939_contig_26474 | ORF2: Phage cluster 79 |
| Ga0209229_10003398 | ORF2: Tail tape measure protein |
| Ga0209229_10007918 | ORF3: Phage cluster 4886 |
| Ga0209229_10008255 | ORF2: Phage cluster 10419 |
| Ga0209229_10012861 | ORF4: Hypothetical protein [PaV-LD] |
| Ga0209229_10017258 | ORF3: Phage cluster 4886 |
| Ga0209229_10021454 | ORF3: Hypothetical protein [PaV-LD] |
| Ga0209229_10026795 | ORF3: Phage cluster 889 |
| Ga0209229_10034259 | ORF1: Hypothetical protein [PaV-LD]  ORF2: Hypothetical protein [PaV-LD]  ORF3: Hypothetical protein [PaV-LD]  ORF4: Hypothetical protein [PaV-LD] |
| 140938_contig_37 | ORF4: Hypothetical protein |
| 140938_contig_1949 | ORF20: DNA polymerase III subunit beta |
| 140939_contig_119 | ORF4: Hypothetical protein |
| 140939_contig_283 | ORF19: Hypothetical protein |
| 140939_contig_345 | ORF25: DNA polymerase III subunit beta |
| Ga0209229_10000210 | ORF11: DNA polymerase III subunit beta |
| Ga0209229_10001252 | ORF18: Hypothetical protein |
| Ga0209229_10001752 | ORF12: Hypothetical protein |
| Ga0209229_10002258 | ORF2: Hypothetical protein |

## Supplementary References

Bullerjahn, G., McKay, R. (2020) Data from Sandusky Bay, Lake Erie from surveys conducted via Ohio Dept of Natural Resources watercraft from June to September 2018. Biological and Chemical Oceanography Data Management Office (BCO-DMO). (Version 1) Version Date 2019-02-07. doi:10.26008/1912/bco-dmo.755348.1
